# Catastrophic Events of Cardiac Sarcoidosis: A Case Report

**DOI:** 10.7759/cureus.24902

**Published:** 2022-05-11

**Authors:** Maria Riasat, Arshan Khan, Moiz Ehtesham, Vineet Meghrajani, Anthony Hafez

**Affiliations:** 1 Internal Medicine, Icahn School of Medicine Mount Sinai Beth Israel, New York, USA; 2 Internal Medicine, Ascension St. John Hospital, Detroit, USA; 3 Internal Medicine, Albany Medical College, Albany, USA; 4 Cardiology, Icahn School of Medicine Mount Sinai Beth Israel, New York, USA; 5 Nuclear Medicine, Icahn School of Medicine at Mount Sinai, New York, USA

**Keywords:** pulmonary sarcoid, fdg-pet scan, atrioventricular block, cardiac arrest, cardiac sarcoidosis

## Abstract

Cardiac sarcoidosis (CS) can be silent in most patients with extrapulmonary sarcoidosis. Atrioventricular (AV) block is the most common clinical presentation, but it can also present as fatal ventricular arrhythmias and sudden cardiac death. Endomyocardial biopsy is the gold standard; however, it is not sensitive since CS can involve the myocardium in a patchy distribution.

Our case depicts a female who presented with syncope; however, her hospital course was complicated by multiple cardiac arrests. Her initial laboratory tests, including an autoimmune workup, were unremarkable. Cardiac magnetic resonance and fluorodeoxyglucose (FDG) positron emission tomography (PET) imaging revealed intramyocardial delayed enhancement of the basal anteroseptal (non-ischemic distribution) and patchy foci of increased uptake in the anteroseptal and inferior myocardial region, respectively. The patient was started on intravenous methylprednisolone, and her condition slowly improved. Post-discharge, the patient followed in the outpatient clinic with a repeat FDG-PET scan revealing resolution of myocardial FDG uptake. She also underwent bronchoscopy with lymph node biopsy showing granulomas and endobronchial biopsy confirming pulmonary sarcoidosis.

## Introduction

Sarcoidosis has been a diagnostic dilemma for physicians mainly because of its heterogeneous manifestations among different groups. It can affect solitary or multiple organs, and its presentation varies depending on the organ system involved. It is characterized in histology by the presence of non-caseating granulomas. Of patients with pulmonary sarcoidosis, 30% have extrapulmonary sarcoidosis. Cardiac sarcoidosis (CS) is one of the organ systems that can be affected. Although not as common as pulmonary sarcoidosis, CS has been seen to affect 5% of patients with systemic sarcoidosis [1-4]. This article discusses CS, including its presentation, diagnosis, and management.

## Case presentation

We present a case of a 42-year-old female with a past medical history of syncope and palpitations. On the morning of admission, the patient experienced sudden onset of palpitations, and syncope worsened after getting up. Symptoms were persistent, which prompted her to call emergency medical services (EMS). She was found to have a heart rate of 30 bpm, and an electrocardiogram (ECG) done by EMS was worrisome for a 3:1 atrioventricular (AV) block.

**Figure 1:**
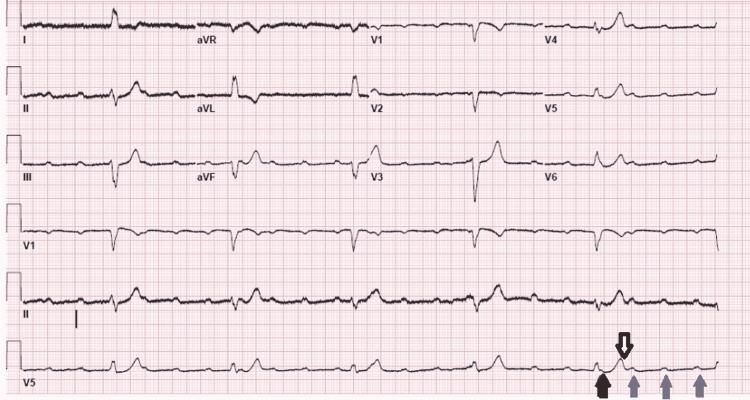
Complete atrioventricular block with QRS shown with a black arrow, T wave with a white arrow, and P waves with blue arrows

The patient was taken to the emergency room (ER) and given atropine 0.5 mg intravenously (IV). On arrival at the ER, the patient was asymptomatic. Initial vital signs included a blood pressure of 113/84 mmHg, a heart rate of 50 beats/minute, a temperature of 98°F, and a respiratory rate of 18 breaths/minute. The rest of the physical examination was unremarkable. Overnight, the patient suddenly became hypotensive and nauseous with a sudden loss of consciousness. The bedside cardiac monitor revealed asystole; the code blue team started chest compressions but stopped when the patient immediately regained consciousness. ECG revealed a complete heart block (Figure 1).

The patient was moved to the cardiac care unit (CCU), before which she had another episode of cardiac arrest, now with hypotension. The patient was started on a peripheral dopamine infusion and had a temporary transvenous pacemaker placed. A transthoracic echocardiogram revealed normal left and right ventricular systolic function with an ejection fraction of 66%. Initial laboratory results are presented in Table 1.

**Table 1:** Laboratory workup

Test	Results	Reference range
White blood count (WBC)	5.5	5.00-11.00 x10E3/uL
Hemoglobin	14	12.0-15.0 G/DL
Platelet	178	150-400 x10E3/uL
Sodium	137	135-145 mmol/L
Potassium	4.7	3.5-5.2 mmol/L
Phosphorus	2.5	2.4-4.7 mg/dL
Magnesium	1.8	1.5-2.5 mg/dL
Creatinine	0.96	0.5-1.1 mg/dL
Blood urea nitrogen	23	6-23 mg/dL
Brain natriuretic peptide (BNP)	50.1	<101 pg/mL
Troponin	0.01	<0.031 mg/dl
Aspartate aminotransferase	17	1-35 U/L
Alanine aminotransferase	12	1-45 U/L
Alkaline phosphatase	43	38-126 U/L
Bilirubin direct	0.5	0.0-0.8 mg/dL
Bilirubin total	1.1	0.1-1.2 mg/dL
C-reactive protein	1.27	<5.1 mg/L
Erythrocyte sedimentation rate	8	0-24 mm/hr
Thyroid-stimulating hormone	0.37	0.40-4.20 uIU/mL
Angiotensin-converting enzyme	29	14-82 U/L
Lyme total antibody test IgG/IgM	Negative	Negative < 0.91; equivocal: 0.91-1.09; positive > 1.09
Antinuclear antibody	Negative	Negative < 1:80; borderline: 1:80; positive > 1:80
Rheumatoid factor	<15	0-15 IU/mL

**Figure 2:**
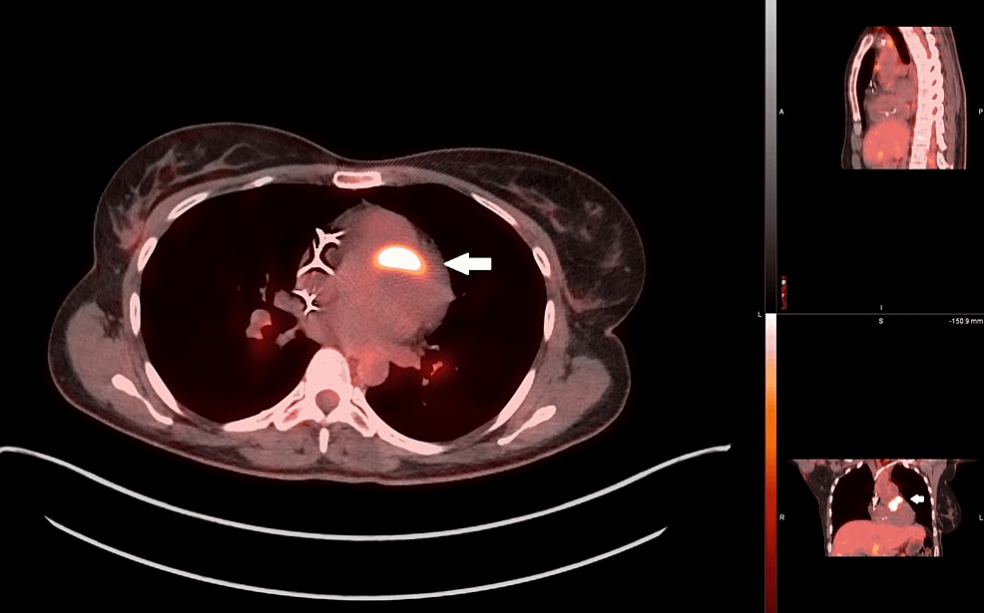
Cardiac PET scan reveals FDG avidity in the anteroseptal region of the myocardium FDG: fluorodeoxyglucose; PET: positron emission tomography.

Blood cultures and respiratory viral panels were negative. Chest X-ray revealed bilateral clear lung fields. Computed tomography (CT) of the chest was negative for lymphadenopathy but revealed an incidental finding of pulmonary embolus in the subsegmental branches. The patient was started on rivaroxaban 15 mg twice daily. Since all her laboratory workup was negative, the patient underwent cardiovascular magnetic resonance (CMR) imaging, which revealed intramyocardial delayed enhancement of the basal anteroseptal (non-ischemic distribution) suggesting inflammatory processes such as sarcoidosis or myocarditis; there was no evidence of myocardial edema. She subsequently underwent fluorodeoxyglucose (FDG) positron emission tomography (PET) (Figure 2), which revealed multiple foci of increased uptake in the anteroseptal and inferior myocardial regions and cervical and mediastinal lymph nodes.

**Figure 3:**
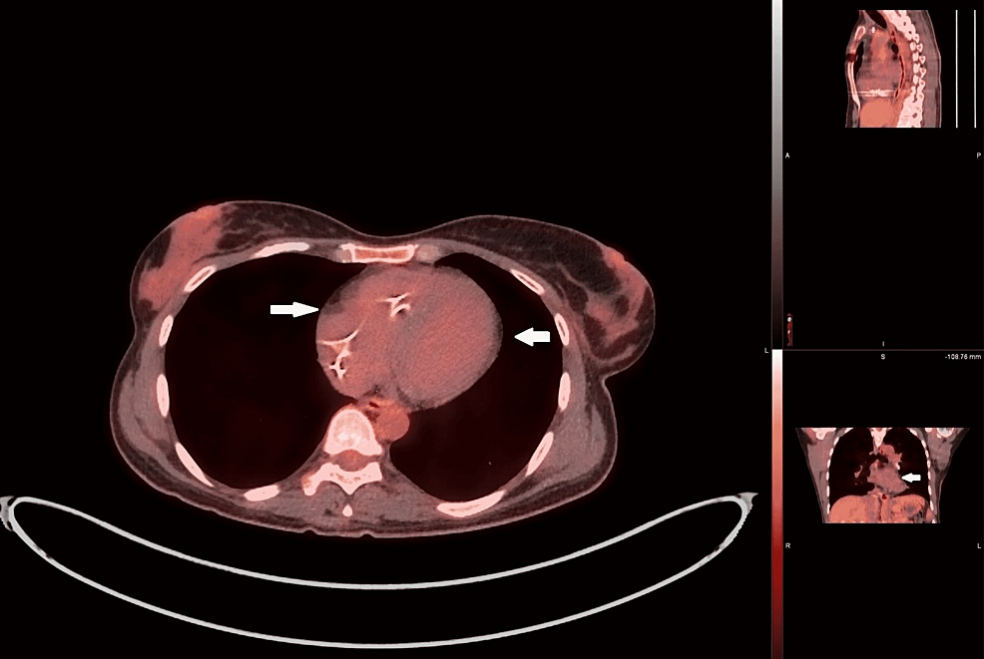
Cardiac PET scan six months post-treatment initiation reveals resolution of FDG-PET uptake as highlighted by white arrows FDG: fluorodeoxyglucose; PET: positron emission tomography.

The patient was started on IV methylprednisolone (Solu-Medrol) 1,000 mg daily. Subsequently, the patient had a permanent pacemaker implanted and was discharged home in stable condition on prednisone 5 mg tablet, methotrexate 2.5 mg, and rivaroxaban 20 mg daily. After six months, repeated FDG-PET in an outpatient setting revealed resolution of myocardial FDG uptake favoring response to medical treatment (Figure 3). Three months after discharge, she underwent bronchoscopy with lymph node biopsy showing granulomas and endobronchial biopsy, which confirmed sarcoidosis involving the lungs and intrathoracic lymph nodes.

## Discussion

Sarcoidosis is an autoimmune disease that can involve multiple organ systems. CS is often diagnosed in patients presenting with pulmonary manifestation; isolated CS is an underdiagnosed disease [1]. Sarcoidosis occurs worldwide and affects any age, gender, and race. The exact burden of disease is difficult to estimate due to vague diagnostic criteria and variability in disease presentation. Sarcoidosis tends to be more severe in African Americans. It is more common in men aged 30-50 years and females aged 50-60 years. Non-caseating granulomas are pathognomonic features of the disease. The disease has variable presentation depending on the organ system that it involves. Several studies have supported the existence of genetic predisposition to develop sarcoidosis, mainly because of familial clustering, increased concordance in monozygotic twins, large variation in frequency, expression, and outcomes in different ethnicities, and presence of certain alleles in the various manifestations of sarcoidosis; one example being that HLA-DRB1*03 allele is strongly associated with Lofgren’s syndrome. Environmental triggers, both organic and inorganic, combined with immune dysregulation are associated with the pathogenesis of sarcoidosis [2,3].

CS can be silent in most patients when associated with extrapulmonary sarcoidosis or may present as palpitations [4]. AV block is the most common clinical presentation in patients with clinically evident CS. Heart failure is another manifestation; however, more fatal manifestations of CS are ventricular tachyarrhythmias that can present as syncope or sudden cardiac death.

Endomyocardial biopsy is specific; however, it is not sensitive since CS can involve the myocardium in a patchy distribution. Some serum biomarkers have been proposed but are not sensitive or specific for diagnosis. Without extracardiac manifestations, the identification of CS remains a diagnostic dilemma. Routine tests like electrocardiogram and transthoracic echocardiogram also lack sensitivity and specificity. CMR imaging is the next step in the diagnostic algorithm, and late gadolinium enhancement (LGE) uptake is usually a trigger for further workup, which includes FDG-PET. FDG-PET is extremely useful as an initial diagnostic test as well as for therapy guidance and can be repeated six months post-treatment initiation. Three sets of criteria have been established for the diagnosis of CS since it remains a challenging diagnosis, i.e., the World Association of Sarcoidosis and other Granulomatous Disorders (WASOG) organ assessment tool, which was published in 2014 and updated in the recent American Thoracic Society guidelines for sarcoidosis diagnosis, Japanese Ministry of Health & Welfare (JMHW), and the Heart Rhythm Society (HRS) criteria. A recent study compared three main diagnostic criteria with a conclusion of low concordance between the Japanese Circulation Society (JCS) criteria and the other two criteria (WASOG and HRS), raising questions about the specificity of the diagnosis of sarcoidosis in the absence of a confirmatory biopsy. Compared with the JMHW criteria, HRS criteria are thought to have greater sensitivity because these criteria include cardiac PET, PET, and MRI, as well as responsiveness to corticosteroid therapy [2,4-11].

Our case depicts a female who presented with syncope. However, her hospital course was complicated by multiple cardiac arrests. Her initial laboratory tests were negative, including an autoimmune workup. The autoimmune disease like sarcoidosis remained high on her differential because of her young age, background, and presence of AV block, which according to the HRS expert consensus statement in 2014 was listed under clinical diagnosis criteria; when found, it increases the probability of CS [8]. Isolated CS was suspected, for which CMR and FDG-PET were done, which revealed intramyocardial delayed enhancement of the basal anteroseptal (non-ischemic distribution) and patchy foci of increased uptake in the anteroseptal and inferior myocardial region (Figure 2), respectively. This was confirmed as CS with pulmonary involvement when the patient later underwent bronchoscopy with lymph node biopsy revealing granulomas and endobronchial biopsy confirming pulmonary sarcoidosis.

Corticosteroid therapy has been the first-line therapy to treat CS for decades [6]. A retrospective study [12] revealed that corticosteroid treatment led to a reduction in ventricular tachycardia, reversal of AV block, improvement in left ventricular ejection fraction, and survival benefits. However, steroid-sparing treatments are also available, methotrexate being the most common second-line agent used. Other second-line therapies include mycophenolate mofetil, azathioprine, cyclophosphamide, and infliximab [13-17]. As mentioned above, treatment response can be guided by a repeat FDG-PET scan, which was done in our patient, revealing decreased FDG uptake (Figure 3).

Pulmonary sarcoidosis and CS are associated with significant morbidity and mortality [2]. Sudden cardiac death is considered the major cause of death in CS. According to the HRS guidelines, patients with identified conduction abnormalities like AV block device implantation along with immunosuppressive therapy are helpful [15].

## Conclusions

CS is rare but should be considered in young patients with heart failure, new AV block, and cardiac arrest, even without pulmonary manifestations of sarcoidosis. Its timely diagnosis is crucial because of its clinical sequelae, including fatal ventricular arrhythmias and sudden cardiac death.
